# The Effectiveness of Collaborative Care Interventions for the Management of Patients With Multimorbidity: Protocol for a Systematic Review, Meta-Analysis, and Meta-Regression Analysis

**DOI:** 10.2196/58296

**Published:** 2024-08-08

**Authors:** Anne-Maj Knudsen, Ann-Cathrine Dalgård Dunvald, Stine Hangaard, Ole Hejlesen, Thomas Kronborg

**Affiliations:** 1 Department of Health Science and Technology Aalborg University Gistrup Denmark; 2 Steno Diabetes Center North Denmark Aalborg University Hospital Aalborg Denmark; 3 Department of Clinical Pharmacology Odense University Hospital Odense Denmark; 4 Department of Clinical Research University of Southern Denmark Odense Denmark

**Keywords:** multimorbidity, comorbidity, multiple chronic conditions, patient care team, multidisciplinary teams, collaborative care, quality of life, systematic review, meta-analysis

## Abstract

**Background:**

Collaborative care interventions have been proposed as a promising strategy to support patients with multimorbidity. Despite this, the effectiveness of collaborative care interventions requires further evaluation. Existing systematic reviews describing the effectiveness of collaborative care interventions in multimorbidity management tend to focus on specific interventions, patient subgroups, and settings. This necessitates a comprehensive review that will provide an overview of the effectiveness of collaborative care interventions for adult patients with multimorbidity.

**Objective:**

This systematic review aims to systematically assess the effectiveness of collaborative care interventions in comparison to usual care concerning health-related quality of life (HRQoL), mental health, and mortality among adult patients with multimorbidity.

**Methods:**

Randomized controlled trials evaluating collaborative care interventions designed for adult patients (18 years and older) with multimorbidity compared with usual care will be considered for inclusion in this review. HRQoL will be the primary outcome. Mortality and mental health outcomes such as rating scales for anxiety and depression will serve as secondary outcomes. The systematic search will be conducted in the CENTRAL, PubMed, CINAHL, and Embase databases. Additional reference and citation searches will be performed in Google Scholar, Web of Science, and Scopus. Data extraction will be comprehensive and include information about participant characteristics, study design, intervention details, and main outcomes. Included studies will be assessed for limitations according to the Cochrane Risk of Bias tool. Meta-analysis will be conducted to estimate the pooled effect size. Meta-regression or subgroup analysis will be undertaken to explore if certain factors can explain the variation in effect between studies, if feasible. The certainty of evidence will be evaluated using the GRADE (Grading of Recommendations Assessment, Development, and Evaluation) approach.

**Results:**

The preliminary literature search was performed on February 16, 2024, and yielded 5255 unique records. A follow-up search will be performed across all databases before submission. The findings will be presented in forest plots, a summary of findings table, and in narrative format. This systematic review is expected to be completed by late 2024.

**Conclusions:**

This review will provide an overview of pooled estimates of treatment effects across HRQoL, mental health, and mortality from randomized controlled trials evaluating collaborative care interventions for adults with multimorbidity. Furthermore, the intention is to clarify the participant, intervention, or study characteristics that may influence the effect of the interventions. This review is expected to provide valuable insights for researchers, clinicians, and other decision-makers about the effectiveness of collaborative care interventions targeting adult patients with multimorbidity.

**Trial Registration:**

International Prospective Register of Systematic Reviews (PROSPERO) CRD42024512554; https://www.crd.york.ac.uk/PROSPERO/display_record.php?RecordID=512554

**International Registered Report Identifier (IRRID):**

DERR1-10.2196/58296

## Introduction

Multimorbidity lacks a universally accepted standard definition but is most commonly defined as the presence of 2 or more coexisting chronic conditions in one individual [1]. Multimorbidity is distinguished from the related concept of comorbidity by treating all conditions equally as opposed to prioritizing or designating an index condition in the case of comorbidity [1].

Multimorbidity is highly prevalent on a global scale [2,3]; however, the prevalence shows substantial variation across studies conducted in different countries and age groups [4]. Approximately 37% of adults worldwide are estimated to have multimorbidity [3]. The number of patients with multimorbidity has been rising worldwide and is expected to continue to increase in the future [3,5]. This is partly due to changing lifestyle risk factors and the aging population [3]. A range of lifestyle factors are associated with a higher risk of developing multimorbidity, such as smoking, excessive alcohol intake, lack of physical activity, obesity, and poor diet quality [6-8]. The prevalence of multimorbidity also commonly increases with age [9] and the majority of patients with multimorbidity are typically 65 years and older [2,10]. However, multimorbidity is also associated with gender [9,10], socioeconomic status [9], and educational level [10,11].

Multimorbidity leads to increased resource use due to a higher health care demand and treatment complexities. Patients with multimorbidity face a higher likelihood of hospital admissions, extended hospital stays, and premature mortality compared to those with a single condition [12,13]. The economic burden of managing patients with multimorbidity is substantial and increases in proportion to the number of additional conditions [14-17]. Multimorbidity is also associated with an increased risk of mental disorder, psychological distress [18], impaired quality of life [19-21], and poorer physical function [22]. Depression is especially common among patients with multimorbidity, being 2-3 times more common than among people with no or only one chronic condition [23]. Thus, multimorbidity negatively impacts quality of life [21].

Collaboration between sectors, specialties, and professions has been proposed as a promising approach to improve health care for patients with multimorbidity. Typically, the health care teams involved in these interventions are composed of a range of different types of professionals (eg, nurses, physicians, or social workers) or professionals from different disciplines or specialties (eg, general practice or endocrinology) [24]. Despite previous systematic reviews exploring the effects of using collaborative care interventions in the management of patients with multimorbidity [25-33], these reviews have generally been limited to a certain type of intervention, subpopulation, or setting. The general findings in these systematic reviews are further subject to uncertainties regarding the effectiveness of collaborative care interventions for managing multimorbidity due to mixed results, insufficient data, and evidence gaps [25-28,31]. Multiple explanations have been suggested for these findings. The current definitions of multimorbidity result in the inclusion of a substantial number of diverse patients, some of whom may not require collaborative care interventions [34]. One plausible explanation is that the effect of collaborative care interventions depends on the target subpopulation, implying that studies involving patients with limited room for improvement demonstrate less effectiveness [25,35]. This emphasizes the importance of identifying the patients who would generally benefit the most from collaborative care interventions.

Collectively, this situation leads to a fragmented overview of the collaborative care interventions used for patients with multimorbidity, necessitating a comprehensive review of these interventions and their corresponding effects. This calls for a new systematic review with a meta-analysis incorporating specific analyses such as meta-regression or subgroup analysis that is capable of handling differences between studies with regard to participant and intervention characteristics. This need is underlined by the rapid development in research within the field of collaborative care interventions for patients with multimorbidity.

Therefore, this systematic review aims to assess the effectiveness of collaborative care interventions compared with usual care on health-related quality of life (HRQoL), mental health, and mortality among adult patients with multimorbidity.

The main research question is: What is the effectiveness of being assigned to collaborative care interventions compared to usual care for adult patients with multimorbidity?

The secondary research question is: Can differences in effectiveness between studies be explained by participant, intervention, or study characteristics?

## Methods

### Study Design

The development of this protocol is based on the PRISMA-P (Preferred Reporting Items for Systematic Review and Meta-Analysis Protocols) checklist [36]. The proceeding systematic review will be conducted according to the PRISMA (Preferred Reporting Items for Systematic Reviews and Meta-Analyses) guidelines [37]. This study was registered on the International Prospective Register of Systematic Reviews (PROSPERO; CRD42024512554) on February 15, 2024.

### Eligibility Criteria

#### Participants

Participants must be adults, defined as being at minimum 18 years of age. The included participants must have multimorbidity, defined as at least 2 chronic conditions in one individual [38]; chronic diseases will be considered according to the World Health Organization definition of “health problems that require ongoing management over a period of years or decades” [39]. Studies with subgroups of patients with multimorbidity, presented in separate subgroup analyses, will be considered for inclusion.

#### Intervention

The definition of collaborative care is based on the systematic review by Gunn et al [40]. The intervention must involve any type of collaborative care activity in addition to the delivery of patient care. The intervention should include at least one health care professional (HCP) and the collaboration must involve at least 2 different health or social professions (eg, nurses, social workers, or pharmacologists) or similar HCPs from at least 2 different specialties (eg, collaboration between 2 physicians specialized in cardiology and endocrinology). Alternative professions such as “health coordinators” or “clinical care managers” will be considered when these professionals actively contribute as independent participants in the intervention, providing coordination or insight from social or health care perspectives. Furthermore, the intervention must encompass a structured management plan and scheduled patient follow-ups [40].

#### Comparator

The comparison must be usual care. For this review, usual care is defined as the level of collaboration expected as part of normal practice, without explicitly mentioning a higher degree of collaboration, inspired by the study of Pascucci et al [41].

#### Outcomes

Prioritization of outcomes in this systematic review is based on the core outcome set for multimorbidity proposed by Smith et al [42]. The primary outcome is HRQoL, given its highest rank in the core outcome set [42]. The secondary outcomes will cover various elements such as mental health outcomes (encompassing general mental health, depression, and anxiety scales) and mortality due to their status as essential outcomes in the core outcome set [42].

#### Study Design

Randomized controlled trials (RCTs) with a parallel design will be considered in this review. Feasibility studies with an RCT design presenting results on effectiveness will also be considered for inclusion. Other criteria encompass inclusion of articles written in English, Danish, Swedish, and Norwegian. The articles must be available in full text. All studies available up to the submission date will be considered for inclusion. Only peer-reviewed articles will be included.

### Information Sources

The systematic research will be conducted in the following scientific databases: CENTRAL, PubMed (including the MEDLINE database), CINAHL, and Embase. All included articles will undergo reference screening and a citation search to ensure relevant papers are captured. Citation searches of relevant articles will be carried out in Scopus, Web of Science, and Google Scholar.

### Search Strategy

The search strategy was developed by conducting an initial search in CENTRAL and PubMed to identify the relevant literature. The retrieved literature from the initial search was then used to identify relevant keywords that were integrated into the search strategy. The search will be performed through a block search structured according to the Population, Intervention, Comparator, and Outcome (PICO) framework, encompassing terms related to the population and intervention. The search strategy will be tailored to the specific database used. Synonyms, similar terms, and variations in spelling will be considered in the searches. The databases’ built-in search functions will also be used, such as the inherent thesaurus, Boolean operators, and filtering options. The search strategies are planned in collaboration with a research librarian. The complete search strategy used in all databases is provided in Multimedia Appendix 1. Prior to submission, a follow-up search in all databases will be performed to ensure inclusion of all newly published studies.

### Study Records

#### Data Management

Following the search, all references will be uploaded into Zotero version 6.0.30 [43]. The function *merge duplicates* in the *duplet section* will initially be used. Subsequently, the references will be exported to the web-based software Covidence [44], facilitating collaborative inclusion and exclusion of studies.

#### Selection Process

Potential studies will be evaluated for inclusion in this review based on the presented eligibility criteria. Two authors will independently assess all studies from the block search. Initially, the studies will be screened by title and abstract, and those not aligning with the eligibility criteria will be excluded. The remaining studies will undergo a full-text assessment, and only those meeting the eligibility criteria will be included. In case of disagreement, a third author will be involved in the decision-making process. Additional citation and reference searches will be performed by the first author. A PRISMA flow diagram will be provided to ensure transparency in the selection of articles (see Multimedia Appendix 2).

#### Data Collection Process

Data extraction will be performed using a customized Microsoft Excel form. In cases where usable data extraction is hindered due to incomplete representation of study details or results in some articles, the authors of these studies will be contacted for clarification.

### Data Items

The information relevant for this review includes elements of the study design, participant characteristics, intervention details, and main outcomes. The data extracted from the studies will encompass various elements, including author details, publication year, country, study design, baseline characteristics, total sample size, intervention specifics, intervention team, setting, comparator, and study outcomes. A table summarizing the key characteristics of each study will be provided. Concerning the type of participants in the study, a distinction will be made between comorbidity studies, focusing on patients with specific combinations of conditions, and multimorbidity studies, encompassing participants with diverse combinations of conditions [45]. Baseline characteristics will include elements such as age, gender, BMI, or body weight. Details about the interventions will encompass aspects such as the setting, intervention duration and frequency, and the types of professionals and specialties represented in the collaborative care teams. Furthermore, it will be noted whether the intervention incorporates a digital element. The collected data items pertaining to the study outcomes will include details such as outcome scores or values, statistical precision metrics, assessment tool, sample size in each group at the time of assessment, and time of assessment. In the meta-analysis, results related to the latest time of assessment will be collected. The authors of the included studies will be contacted to provide additional information if characteristics or results at the study level are not available.

### Risk of Bias Assessment

The included studies will be assessed for limitations according to the Cochrane Risk of Bias tool version 2 [46], with independent assessments conducted by 2 authors. In cases where differences in assessment occur, a discussion involving a third author will be initiated to reach agreement. All eligible studies will be included in this review, regardless of their quality. Only studies with an overall risk of bias judged to be “low risk of bias” or “some concerns” will be included in the meta-analysis. If missing information makes risk of bias assessment impossible, corresponding authors will be contacted to obtain the necessary information. The results of the risk of bias assessment will be presented in a risk of bias chart.

### Data Synthesis

#### Meta-Analysis

A meta-analysis will be performed to analyze the available data. The analysis will be performed in RStudio using the Meta-Analysis Package for R (Metafor package) enabling the creation of a data frame containing various measures for the meta-analysis model (eg, effect size, *t* statistics, or *P* values). However, if the measures differ due to discrepancies in the data provided by some studies, supplementary calculations to standardize the data for analysis will be performed. These adjustments will adhere to the equations outlined in the Cochrane Handbook [47]. Outcome scores or values given as the absolute mean difference from baseline or absolute mean at follow-up, along with statistical precision metrics (eg, SD, CI, or SE), will be used to estimate the overall effect size for continuous variables. For studies where these data are not reported, the between-group estimates that quantify the effect of the intervention on the outcome and their precision will be used. If results are presented as medians and IQRs, the means and SDs will be estimated [48]. The article with the largest sample will be used for the meta-analysis if 2 or more articles present data from the same study for a particular outcome.

The meta-analysis will be based on the standardized mean difference for continuous variables sharing the same construct but different outcome measures. There is no rule as to when certain outcomes measures can be combined, which is generally determined by clinical judgment [49]. The decision to combine different outcome measures will involve at least 2 authors. Should disagreement arise, a third author will be consulted to reach agreement. Conversely, the weighted mean difference will be applied for continuous variables sharing the same outcome measure.

As it is difficult to make assumptions about the underlying distribution of effect sizes, the meta-analysis will follow the pragmatic approach proposed in the Cochrane Handbook regarding the choice of model [50]. This means that both the random- and fixed-effects models will be used in this meta-analysis because the included studies are expected to vary in multiple parameters [51]. One parameter is the samples in the studies, which do not originate from the same population. Although the overall population is patients with multimorbidity, the selected samples in the studies are expected to vary considerably. Consequently, the effects estimated across the studies may not be identical but rather follow a similar distribution. The findings from both models will be presented using forest plots and tables, accompanied by a sensitivity analysis. The effect estimate of continuous variables will be presented as a pooled mean difference or pooled standardized mean difference with 95% CIs. Odds ratios will be given for dichotomous variables with 95% CIs. Two-sided *P* values <.05 will be considered statistically significant. Interpretation of the pooled effect size will be guided by the principles outlined in the Cochrane Handbook [52]. To test for heterogeneity, the Higgins *I*^2^ statistic will be calculated. The interpretation of *I*^2^ will also be made according to the Cochrane Handbook [50].

#### Sensitivity Analysis

Sensitivity analysis will be performed, including variations in statistical models (random vs fixed effects) as well as assessments of risk of bias. If relevant, sensitivity analysis will also be performed by excluding studies that only report medians and IQRs. Other potential sensitivity analyses may consider a range of parameters such as sample size or follow-up length, with the purpose of evaluating the potential impact of these factors on the overall findings. A summary table will showcase the results of any sensitivity analyses performed [50].

#### Meta-Regression and Subgroup Analysis

Meta-regression or subgroup analysis will be performed to explore if certain factors can explain the variation in effect between studies. To explore associations between the primary outcome and various factors in the meta-analysis, a meta-regression analysis will be performed. In the analysis, participant characteristics will be examined by distinguishing between comorbidity and multimorbidity studies. Other participant-related factors to be explored include demographics, socioeconomic status, health indicators, lifestyle factors, and treatment variables. Interventions will be examined across multiple dimensions, including setting, duration, and frequency. Additionally, the types of professionals and specialties represented in the collaborative care teams will be evaluated to understand their influence on intervention outcomes. Furthermore, the presence of digital components will be considered to assess if they contribute to variations in effectiveness across studies.

Subgroup analysis may be performed if the meta-regression is impossible due to limitations related to the data. This may be due to the limited availability of continuous variables if studies predominantly report variables categorically (eg, in case of age groups instead of age as a continuous variable). Additionally, meta-regression analysis will not be considered if there are fewer than 10 studies in a meta-analysis [50].

#### Narrative Format

A table containing a narrative description of the key characteristics and findings of all studies will be provided. Due to heterogeneity or high risk of bias, meta-analysis may not be feasible for certain outcomes. In these cases, a narrative description will be provided. The results of the quantitative synthesis will be presented in a table with a summary of findings.

### Assessment of Meta-Biases

To assess potential publication biases, a funnel plot with a regression line will be created. The funnel plot will be complemented by a statistical test for funnel plot asymmetry, such as the Egger test. The funnel plot will only be constructed for evaluation if the specific meta-analysis contains results from at least 10 studies [53].

### Confidence in Cumulative Evidence

The GRADE (The Grading of Recommendations Assessment, Development, and Evaluation) system will be used for grading the certainty of evidence [54]. The GRADE evaluation will be performed independently by 2 authors. In case of disagreement, a third author will perform an additional GRADE evaluation. The web-based GRADEpro Guideline Development Tool software (Evidence Prime) will be used to perform the evaluation [55]. The results from the GRADE evaluation will be presented in a summary of findings table. This grading will be based on risk of bias, directness, heterogeneity, precision, and risk of publication bias.

## Results

This systematic review and meta-analysis is ongoing. Results from the preliminary search, performed on February 16, 2024, can be found in Multimedia Appendices 1 and 2. A total of 6730 records were retrieved, of which 1475 were duplicates. This resulted in 5255 unique records remaining for initial title and abstract screening. A follow-up search across all databases will be conducted prior to submission. The findings will be visually presented through forest plots and in a summary of findings table where meta-analysis is feasible, and in cases where it is not, through narrative comparisons. This systematic review and meta-analysis is expected to be completed in late 2024. Following completion, the results will undergo peer review before being submitted for publication.

## Discussion

This systematic review aims to assess the treatment effect on HRQoL, mental health, and mortality in collaborative care interventions compared to usual care in adult patients with multimorbidity. The presented systematic review will have both strengths and limitations. The strengths are rooted in the comprehensive exploration and broad scope undertaken. This review aims to investigate the impact of heterogeneity in participant and intervention characteristics on the pooled effect size. Such exploration holds the potential to provide valuable insights into the effectiveness of collaborative care interventions and to identify the factors that influence this effect. This may contribute to determining the optimal selection of patients with multimorbidity expected to derive the greatest benefit from collaborative care interventions. Additionally, this review may reveal intervention characteristics that prove meaningful to investigate further. Conducting a subsequent qualitative analysis may offer a deeper understanding of aspects that quantitative data alone may not capture. Triangulating these findings therefore ensures a thorough investigation of the complexities inherent in collaborative care interventions for patients with multimorbidity.

Nevertheless, limitations are expected due to the high degree of clinical diversity. This variability extends across the population, encompassing diverse conditions and diagnoses, as well as within the intervention characteristics, where differences stem from factors such as the composition of the collaborative care teams and the settings where the interventions are implemented. The planned comparison is therefore expected to be complicated due to the lack of homogeneity within the field. Hence, the forthcoming discussion will also encompass the consideration of confounders and methodological limitations to ensure meaningful conclusions. Further limitations will be examined and elucidated in the discussion section of the review to contribute valuable insights for guiding future research efforts.

## Appendix

Multimedia Appendix 1 Search strategy.

Multimedia Appendix 2 Study selection flow diagram.

## Abbreviations

GRADE: Grading of Recommendations Assessment, Development, and Evaluation
HCP: health care professional
HRQoL: health-related quality of life
PICO: patient, intervention, comparison, outcome
PRISMA: Preferred Reporting Items for Systematic Reviews and Meta-Analyses
PRISMA-P: Preferred Reporting Items for Systematic Review and Meta-Analysis Protocols
PROSPERO: International Prospective Register of Systematic Reviews
RCT: randomized controlled trial

## References

[1] Valderas JM, Starfield B, Sibbald B, Salisbury C, Roland M (2009). Defining comorbidity: implications for understanding health and health services. Ann Fam Med.

[2] Nguyen H, Manolova G, Daskalopoulou C, Vitoratou S, Prince M, Prina AM (2019). Prevalence of multimorbidity in community settings: a systematic review and meta-analysis of observational studies. J Comorb.

[3] Chowdhury SR, Chandra Das D, Sunna TC, Beyene J, Hossain A (2023). Global and regional prevalence of multimorbidity in the adult population in community settings: a systematic review and meta-analysis. EClinicalMedicine.

[4] Salive ME (2013). Multimorbidity in older adults. Epidemiol Rev.

[5] Uijen AA, van de Lisdonk EH (2008). Multimorbidity in primary care: prevalence and trend over the last 20 years. Eur J Gen Pract.

[6] Katikireddi SV, Skivington K, Leyland AH, Hunt K, Mercer SW (2017). The contribution of risk factors to socioeconomic inequalities in multimorbidity across the lifecourse: a longitudinal analysis of the Twenty-07 cohort. BMC Med.

[7] Freisling H, Viallon V, Lennon H, Bagnardi V, Ricci C, Butterworth AS, Sweeting M, Muller D, Romieu I, Bazelle P, Kvaskoff M, Arveux P, Severi G, Bamia C, Kühn T, Kaaks R, Bergmann M, Boeing H, Tjønneland A, Olsen A, Overvad K, Dahm CC, Menéndez V, Agudo A, Sánchez MJ, Amiano P, Santiuste C, Gurrea AB, Tong TYN, Schmidt JA, Tzoulaki I, Tsilidis KK, Ward H, Palli D, Agnoli C, Tumino R, Ricceri F, Panico S, Picavet HSJ, Bakker M, Monninkhof E, Nilsson P, Manjer J, Rolandsson O, Thysell E, Weiderpass E, Jenab M, Riboli E, Vineis P, Danesh J, Wareham NJ, Gunter MJ, Ferrari P (2020). Lifestyle factors and risk of multimorbidity of cancer and cardiometabolic diseases: a multinational cohort study. BMC Med.

[8] Fortin M, Haggerty J, Almirall J, Bouhali T, Sasseville M, Lemieux M (2014). Lifestyle factors and multimorbidity: a cross sectional study. BMC Public Health.

[9] Violan C, Foguet-Boreu Q, Flores-Mateo G, Salisbury C, Blom J, Freitag M, Glynn L, Muth C, Valderas JM (2014). Prevalence, determinants and patterns of multimorbidity in primary care: a systematic review of observational studies. PLoS One.

[10] Frølich A, Ghith N, Schiøtz M, Jacobsen R, Stockmarr A (2019). Multimorbidity, healthcare utilization and socioeconomic status: a register-based study in Denmark. PLoS One.

[11] Pathirana TI, Jackson CA (2018). Socioeconomic status and multimorbidity: a systematic review and meta-analysis. Aust N Z J Public Health.

[12] Menotti A, Mulder I, Nissinen A, Giampaoli S, Feskens EJ, Kromhout D (2001). Prevalence of morbidity and multimorbidity in elderly male populations and their impact on 10-year all-cause mortality: The FINE study (Finland, Italy, Netherlands, Elderly). J Clin Epidemiol.

[13] Vogeli C, Shields AE, Lee TA, Gibson TB, Marder WD, Weiss KB, Blumenthal D (2007). Multiple chronic conditions: prevalence, health consequences, and implications for quality, care management, and costs. J Gen Intern Med.

[14] Picco L, Achilla E, Abdin E, Chong SA, Vaingankar JA, McCrone P, Chua HC, Heng D, Magadi H, Ng LL, Prince M, Subramaniam M (2016). Economic burden of multimorbidity among older adults: impact on healthcare and societal costs. BMC Health Serv Res.

[15] Wang L, Si L, Cocker F, Palmer AJ, Sanderson K (2018). A systematic review of cost-of-illness studies of multimorbidity. Appl Health Econ Health Policy.

[16] Tran PB, Kazibwe J, Nikolaidis GF, Linnosmaa I, Rijken M, van Olmen J (2022). Costs of multimorbidity: a systematic review and meta-analyses. BMC Med.

[17] Lehnert T, Heider D, Leicht H, Heinrich S, Corrieri S, Luppa M, Riedel-Heller S, König HH (2011). Review: Health care utilization and costs of elderly persons with multiple chronic conditions. Med Care Res Rev.

[18] Fortin M, Bravo G, Hudon C, Lapointe L, Dubois M, Almirall J (2006). Psychological distress and multimorbidity in primary care. Ann Fam Med.

[19] Fortin M, Lapointe L, Hudon C, Vanasse A, Ntetu AL, Maltais D (2004). Multimorbidity and quality of life in primary care: a systematic review. Health Qual Life Outcomes.

[20] Fortin M, Bravo G, Hudon C, Lapointe L, Almirall J, Dubois M, Vanasse A (2006). Relationship between multimorbidity and health-related quality of life of patients in primary care. Qual Life Res.

[21] Makovski TT, Schmitz S, Zeegers MP, Stranges S, van den Akker M (2019). Multimorbidity and quality of life: systematic literature review and meta-analysis. Ageing Res Rev.

[22] Kadam UT, Croft PR, North Staffordshire GP Consortium Group (2007). Clinical multimorbidity and physical function in older adults: a record and health status linkage study in general practice. Fam Pract.

[23] Read JR, Sharpe L, Modini M, Dear BF (2017). Multimorbidity and depression: a systematic review and meta-analysis. J Affect Disord.

[24] Chamberlain-Salaun J, Mills J, Usher K (2013). Terminology used to describe health care teams: an integrative review of the literature. J Multidiscip Healthc.

[25] Smith SM, Wallace E, Clyne B, Boland F, Fortin M (2021). Interventions for improving outcomes in patients with multimorbidity in primary care and community setting: a systematic review. Syst Rev.

[26] Li M, Tang H, Liu X (2023). Primary care team and its association with quality of care for people with multimorbidity: a systematic review. BMC Prim Care.

[27] Lammila-Escalera E, Greenfield G, Barber S, Nicholls D, Majeed A, Hayhoe BWJ (2022). A systematic review of Interventions that use multidisciplinary team meetings to manage multimorbidity in primary care. Int J Integr Care.

[28] Kappelin C, Carlsson AC, Wachtler C (2022). Specific content for collaborative care: a systematic review of collaborative care interventions for patients with multimorbidity involving depression and/or anxiety in primary care. Fam Pract.

[29] Musuuza J, Sutherland BL, Kurter S, Balasubramanian P, Bartels CM, Brennan MB (2020). A systematic review of multidisciplinary teams to reduce major amputations for patients with diabetic foot ulcers. J Vasc Surg.

[30] Huang Y, Wei X, Wu T, Chen R, Guo A (2013). Collaborative care for patients with depression and diabetes mellitus: a systematic review and meta-analysis. BMC Psychiatry.

[31] Helou N, Dwyer A, Shaha M, Zanchi A (2016). Multidisciplinary management of diabetic kidney disease: a systematic review and meta-analysis. JBI Database System Rev Implement Rep.

[32] Di Serafino F, Pascucci D, Sassano M, Di Pilla A, Carini E, Specchia M, Ricciardi W, Damiani G (2020). Systematic review on multidisciplinarity and management of multimorbid chronic patients in hospital. Eur J Public Health.

[33] Acosta-García H, Alfaro-Lara ER, Sánchez-Fidalgo S, Sevilla-Sánchez D, Delgado-Silveira E, Juanes-Borrego A, Santos-Ramos B (2020). Intervention effectiveness by pharmacists integrated within an interdisciplinary health team on chronic complex patients. Eur J Public Health.

[34] Wallace E, Salisbury C, Guthrie B, Lewis C, Fahey T, Smith SM (2015). Managing patients with multimorbidity in primary care. BMJ.

[35] Skou ST, Mair FS, Fortin M, Guthrie B, Nunes BP, Miranda JJ, Boyd CM, Pati S, Mtenga S, Smith SM (2022). Multimorbidity. Nat Rev Dis Primers.

[36] Shamseer L, Moher D, Clarke M, Ghersi D, Liberati A, Petticrew M, Shekelle P, Stewart LA, PRISMA-P Group (2015). Preferred reporting items for systematic review and meta-analysis protocols (PRISMA-P) 2015: elaboration and explanation. BMJ.

[37] Page MJ, McKenzie JE, Bossuyt PM, Boutron I, Hoffmann TC, Mulrow CD, Shamseer L, Tetzlaff JM, Akl EA, Brennan SE, Chou R, Glanville J, Grimshaw JM, Hróbjartsson A, Lalu MM, Li T, Loder EW, Mayo-Wilson E, McDonald S, McGuinness LA, Stewart LA, Thomas J, Tricco AC, Welch VA, Whiting P, Moher D (2021). The PRISMA 2020 statement: an updated guideline for reporting systematic reviews. BMJ.

[38] Integrated Health Services (IHS) WHO Team (2016). Multimorbidity: technical series on safer primary care. World Health Organization.

[39] Management-Screening, Diagnosis and Treatment (MND) WHO Team (2002). Innovative care for chronic conditions: building blocks for actions. World Health Organization.

[40] Gunn J, Diggens J, Hegarty K, Blashki G (2006). A systematic review of complex system interventions designed to increase recovery from depression in primary care. BMC Health Serv Res.

[41] Pascucci D, Sassano M, Nurchis MC, Cicconi M, Acampora A, Park D, Morano C, Damiani G (2021). Impact of interprofessional collaboration on chronic disease management: findings from a systematic review of clinical trial and meta-analysis. Health Policy.

[42] Smith SM, Wallace E, Salisbury C, Sasseville M, Bayliss E, Fortin M (2018). A core outcome set for multimorbidity research (COSmm). Ann Fam Med.

[43] Zotero.

[44] Covidence.

[45] Smith SM, Wallace E, O'Dowd T, Fortin M (2016). Interventions for improving outcomes in patients with multimorbidity in primary care and community settings. Cochrane Database Syst Rev.

[46] Higgins J, Savovic J, Page M, Elbers R, Sterne J (2023). Chapter 8: Assessing risk of bias in a randomized trial. Cochrane Handbook for Systematic Reviews of Interventions version 6.4.

[47] Higgins J PT, Li T, Deeks JJ (2023). Chapter 6: Choosing effect measures and computing estimates of effect. Cochrane Handbook for Systematic Reviews of Interventions version 6.4.

[48] Wan X, Wang W, Liu J, Tong T (2014). Estimating the sample mean and standard deviation from the sample size, median, range and/or interquartile range. BMC Med Res Methodol.

[49] Higgins J, Green S (2011). 9.1.4 When not to use meta-analysis in a review. Cochrane Handbook for Systematic Reviews of Interventions version 5.1.0.

[50] Deeks JJ, Higgins J PT, Altman D G (2023). Chapter 10: Analysing data and undertaking meta-analyses. Cochrane Handbook for Systematic Reviews of Interventions version 6.4.

[51] Spineli LM, Pandis N (2020). Meta-analysis: random-effects model. Am J Orthod Dentofacial Orthop.

[52] Schünemann H, Vist G, Higgins J, Santesso N, Deeks J, Glasziou P, Akl E, Guyatt G (2023). Chapter 15: Interpreting results and drawing conclusions. Cochrane Handbook for Systematic Reviews of Interventions version 6.4.

[53] Page M, Higgins J, Sterne J (2023). Chapter 13: Assessing risk of bias due to missing results in a synthesis. Cochrane Handbook for Systematic Reviews of Interventions version 6.4.

[54] Guyatt G, Oxman AD, Akl EA, Kunz R, Vist G, Brozek J, Norris S, Falck-Ytter Y, Glasziou P, DeBeer H, Jaeschke R, Rind D, Meerpohl J, Dahm P, Schünemann HJ (2011). GRADE guidelines: 1. Introduction-GRADE evidence profiles and summary of findings tables. J Clin Epidemiol.

[55] McMaster University, Evidence Prime Inc (2021). GRADEpro.

