# Supplementary material for: The Effectiveness of Collaborative Care Interventions for the Management of Patients With Multimorbidity: Protocol for a Systematic Review, Meta-Analysis, and Meta-Regression Analysis
Source: JMIR Res Protoc. 2024 Aug 8;13:e58296. doi: 10.2196/58296 (PMC11342003; doi:10.2196/58296)
Supplement: Multimedia Appendix 1 [file resprot_v13i1e58296_app1.docx]

# Multimedia Appendix 1: Search strategy

**PubMed**
(Date of last search: 16/02/2024)

| **Search** | **Query** | **Records retrieved** |
| --- | --- | --- |
| Block 1: Population – multimorbidity | | |
| #1 | “multiple chronic conditions” [MeSH Terms] | 735 |
| #2 | "comorbidity" [MeSH Major Topic] | 5167 |
| #3 | “multimorbidity” [MeSH Terms] | 3140 |
| #4 | "multimorbidity"[Title/Abstract] OR "multiple chronic condition*"[Title/Abstract] OR "concurrent chronic conditions"[Title/Abstract] OR "concurrent chronic diseases"[Title/Abstract] OR "concurrent chronic disorders"[Title/Abstract] OR "concurrent chronic health conditions"[Title/Abstract] OR "concurrent chronic illnesses"[Title/Abstract] OR "concurrent chronic medical conditions"[Title/Abstract] OR comorbidity[Title/Abstract] OR “multiple pathology"[Title/Abstract] OR “polypathology” [Title/Abstract] OR “poly pathology” [Title/Abstract] OR “multipathology” [Title/Abstract] OR “plurimorbidity” [Title/Abstract] OR “multicondition” [Title/Abstract] OR “multiple chronic disorders” [Title/Abstract] OR ”multiple chronic health conditions” [Title/Abstract] OR ”multiple chronic illnesses” [Title/Abstract] OR ”multiple chronic conditions” [Title/Abstract] OR ”multiple chronic medical conditions” [Title/Abstract] | 104,221 |
| #5 | #1 OR #2 OR #3 OR #4 | 106,166 |
| Block 2: Intervention – collaborative care interventions | | |
| #6 | “intersectoral collaboration”[Mesh] | 2584 |
| #7 | “interprofessional relations”[Mesh] | 72,755 |
| #8 | "delivery of health care, integrated"[Mesh] | 14,581 |
| #9 | "patient care team"[Mesh] | 73,131 |
| #10 | “interdisciplinary communication”[Mesh] | 18,151 |
| #11 | "care team*"[Title/Abstract] OR "cross disciplinary"[Title/Abstract] OR "cross-disciplinary"[Title/Abstract] OR “crossdisciplinary” [Title/Abstract] OR "multidisciplinary"[Title/Abstract] OR "multi-disciplinary*"[Title/Abstract] OR "multi disciplinary"[Title/Abstract] OR "interdisciplinary"[Title/Abstract] OR “interdisciplinary*”[Title/Abstract] OR "inter-disciplinary"[Title/Abstract] OR "comprehensive healthcare"[Title/Abstract] OR "continuity of patient care"[Title/Abstract] OR "guided care"[Title/Abstract] OR "integrated care"[Title/Abstract] OR "managed care"[Title/Abstract] OR “care manage*”[Title/Abstract] OR "shared care"[Title/Abstract] OR "integrated healthcare"[Title/Abstract] “collaboratory” [Title/Abstract] OR “collaborative” [Title/Abstract] OR 'transmural care'[Title/Abstract] OR “multiprofessional” [Title/Abstract] OR “multi professional” [Title/Abstract] OR “multi-professional” [Title/Abstract] | 81,666 |
| #12 | #5 OR #6 OR #7 OR #8 OR #9 | 222,749 |
| Block 5: Combined search | | |
| #13 | #4 AND #10 | 1840 |

**Embase**
(Date of last search: 16/02/2024)

| **Search** | **Query** | **Records retrieved** |
| --- | --- | --- |
| Block 1: Population – multimorbidity | | |
| #1 | ‘multiple chronic conditions’/exp | 9187 |
| #2 | ‘comorbidity’/mj | 26,258 |
| #3 | 'multimorbidity':ab,ti OR 'multiple chronic condition*':ab,ti OR 'concurrent chronic conditions':ab,ti OR 'concurrent chronic diseases':ab,ti OR 'concurrent chronic disorders':ab,ti OR 'concurrent chronic health conditions':ab,ti OR 'concurrent chronic illnesses':ab,ti OR 'concurrent chronic medical conditions':ab,ti OR 'multiple pathology':ab,ti OR 'polypathology':ab,ti OR 'poly pathology':ab,ti OR 'multipathology':ab,ti OR 'plurimorbidity':ab,ti OR 'multicondition':ab,ti OR 'multiple chronic disorders':ab,ti OR 'multiple chronic health conditions':ab,ti OR 'multiple chronic illnesses':ab,ti OR 'multiple chronic conditions':ab,ti OR 'multiple chronic medical conditions':ab,ti OR 'simultaneous chronic illnesses':ab,ti OR 'simultaneous chronic medical conditions':ab,ti | 13,509 |
| #4 | #1 OR #2 OR #3 | 41,400 |
| Block 2: Intervention – collaborative care interventions | | |
| #5 | ‘intersectoral collaboration’/exp | 4431 |
| #6 | ‘collaborative care team’/exp | 3391 |
| #7 | ‘interdisciplinary communication’/exp | 13,682 |
| #8 | ‘multidisciplinary team’/exp | 33,820 |
| #9 | 'integrated health care system'/exp | 13,804 |
| #10 | ‘collaborative care’/exp | 109 |
| #11 | 'interdisciplinary collaboration':ab,ti OR 'inter-disciplinary collaboration':ab,ti OR 'inter disciplinary collaboration':ab,ti OR 'care team*':ab,ti OR 'cross disciplinary':ab,ti OR 'cross-disciplinary':ab,ti OR 'inter disciplinary team*':ab,ti OR 'interdisciplinary team*':ab,ti OR 'multi-disciplinary team*':ab,ti OR 'multidisciplinary team*':ab,ti OR 'multidisciplinary collaboration':ab,ti OR 'multi-disciplinary collaboration':ab,ti OR 'multi disciplinary collaboration':ab,ti OR 'comprehensive healthcare':ab,ti OR 'continuity of patient care':ab,ti OR 'patient care management':ab,ti OR 'guided care':ab,ti OR 'integrated care':ab,ti OR 'managed care':ab,ti OR 'care manage*':ab,ti OR 'shared care':ab,ti OR 'delivery of integrated healthcare':ab,ti OR 'collaboratory':ab,ti OR 'collaborative':ab,ti OR 'patient care team':ab,ti OR 'transmural care':ab,ti OR ‘multiprofessional’:ab,ti OR ‘multi professional’:ab,ti OR ‘multi-professional’:ab,ti | 249,237 |
| #12 | #5 OR #6 OR #7 OR #8 OR #9 OR #10 OR #11 | 288,487 |
| Block 5: Combined search | | |
| #13 | #5 AND #12 | 1771 |

**CINAHL**
(Date of last search: 16/02/2024)

| **Search** | **Query** | **Records retrieved** |
| --- | --- | --- |
| Block 1: Population – multimorbidity | | |
| #1 | MJ "comorbidity" | 13,954 |
| #2 | TI "multimorbidity" OR TI "multiple chronic condition*" OR TI "concurrent chronic conditions" OR TI "concurrent chronic diseases" OR TI "concurrent chronic disorders" OR TI "concurrent chronic health conditions" OR TI "concurrent chronic illnesses" OR TI "concurrent chronic medical conditions" OR TI “comorbidity” OR TI “multiple pathology" OR TI “polypathology” OR TI “poly pathology” OR TI “multipathology” OR TI “plurimorbidity” OR TI “multicondition” OR TI “multiple chronic disorders” OR TI ”multiple chronic health conditions” OR TI ”multiple chronic illnesses” OR TI ”multiple chronic conditions” OR TI ”multiple chronic medical conditions” OR AB "multimorbidity" OR AB "multiple chronic condition*" OR AB "concurrent chronic conditions" OR AB "concurrent chronic diseases" OR AB "concurrent chronic disorders" OR AB "concurrent chronic health conditions" OR AB "concurrent chronic illnesses" OR AB "concurrent chronic medical conditions" OR AB “comorbidity” OR AB “multiple pathology" OR AB “polypathology” OR AB “poly pathology” OR AB “multipathology” OR AB “plurimorbidity” OR AB “multicondition” OR AB “multiple chronic disorders” OR AB ”multiple chronic health conditions” OR AB ”multiple chronic illnesses” OR AB ”multiple chronic conditions” OR AB ”multiple chronic medical conditions” | 31,317 |
| #3 | #1 OR #2 | 41,031 |
| Block 2: Intervention – collaborative care interventions | | |
| #4 | MH “interprofessional relations” | 29,415 |
| #5 | MH "multidisciplinary care team" | 52,050 |
| #6 | MH "health care delivery, integrated" | 15,328 |
| #7 | TI "care team*" OR TI "cross disciplinary" OR TI "cross-disciplinary" OR TI “crossdisciplinary” OR TI "multidisciplinary" OR TI "multi-disciplinary" OR TI "multi disciplinary" OR TI "interdisciplinary" OR TI “interdisciplinary*” OR TI "inter-disciplinary" OR TI "comprehensive healthcare" OR TI "continuity of patient care" OR TI "guided care" OR TI "integrated care" OR TI "managed care" OR TI “care manage*” OR TI "shared care" OR TI "integrated healthcare" OR TI “collaboratory” OR TI “collaborative” OR TI “transmural care” OR TI “multiprofessional” OR TI “multi professional” OR TI “multi-professional” OR AB "care team*" OR AB "cross disciplinary" OR AB "cross-disciplinary" OR AB “crossdisciplinary” OR AB "multidisciplinary" OR AB "multi-disciplinary" OR AB "multi disciplinary" OR AB "interdisciplinary" OR AB “interdisciplinary*” OR AB "inter-disciplinary" OR AB "comprehensive healthcare" OR AB "continuity of patient care" OR AB "guided care" OR AB "integrated care" OR AB "managed care" OR AB “care manage*” OR AB "shared care" OR AB "integrated healthcare" OR AB “collaboratory” OR AB “collaborative” OR AB 'transmural care' OR AB “multiprofessional” OR AB “multi professional” OR AB “multi-professional” | 138,113 |
| #8 | #4 OR #5 OR #6 OR #7 | 199,299 |
| Block 5: Combined search | | |
| #9 | #3 AND #8 | 2297 |

**The Cochrane Library (CENTRAL)**
(Date of last search: 16/02/2024)

| **Search** | **Query** | **Records retrieved** |
| --- | --- | --- |
| Block 1: Population – multimorbidity | | |
| #1 | MeSH descriptor: [Multimorbidity] explode all trees | 149 |
| #2 | MeSH descriptor: [Comorbidity] explode all trees | 5257 |
| #3 | MeSH descriptor: [Multiple Chronic Conditions] explode all trees | 80 |
| #4 | Multimorbidity:ti OR multiple NEXT chronic NEXT condition*:ti OR concurrent NEXT chronic NEXT conditions:ti OR concurrent NEXT chronic NEXT diseases:ti OR concurrent NEXT chronic NEXT disorders:ti OR concurrent NEXT chronic NEXT health NEXT conditions:ti OR concurrent NEXT chronic NEXT illnesses:ti OR concurrent NEXT chronic NEXT medical NEXT conditions:ti OR comorbidity:ti OR multiple NEXT pathology:ti OR polypathology:ti OR poly NEXT pathology:ti OR multipathology:ti OR plurimorbidity:ti OR multicondition:ti OR multiple NEXT chronic NEXT disorders:ti OR multiple NEXT chronic NEXT health NEXT conditions:ti OR multiple NEXT chronic NEXT illnesses:ti OR multiple NEXT chronic NEXT conditions:ti OR multiple NEXT chronic NEXT medical NEXT conditions:ti OR multimorbidity:ab OR multiple NEXT chronic NEXT condition*:ab OR concurrent NEXT chronic NEXT conditions:ab OR concurrent NEXT chronic NEXT diseases:ab OR concurrent NEXT chronic NEXT disorders:ab OR concurrent NEXT chronic NEXT health NEXT conditions:ab OR concurrent NEXT chronic NEXT illnesses:ab OR concurrent NEXT chronic NEXT medical NEXT conditions:ab OR comorbidity:ab OR multiple NEXT pathology:ab OR polypathology:ab OR poly NEXT pathology:ab OR multipathology:ab OR plurimorbidity:ab OR multicondition:ab OR multiple NEXT chronic NEXT disorders:ab OR multiple NEXT chronic NEXT health NEXT conditions:ab OR multiple NEXT chronic NEXT illnesses:ab OR multiple NEXT chronic NEXT conditions:ab OR multiple NEXT chronic NEXT medical NEXT conditions:ab | 8484 |
| #5 | #1 OR #2 OR #3 OR #4 | 12,801 |
| Block 2: Intervention – collaborative care interventions | | |
| #6 | MeSH descriptor: [Interdisciplinary Communication] explode all trees | 373 |
| #7 | MeSH descriptor: [Patient Care Team] explode all trees | 2291 |
| #8 | MeSH descriptor: [Delivery of Health Care, Integrated] explode all trees | 585 |
| #9 | MeSH descriptor: [Intersectoral Collaboration] explode all trees | 81 |
| #10 | MeSH descriptor: [Interprofessional Relations] explode all trees | 812 |
| #11 | care NEXT team*:ti OR cross NEXT disciplinary:ti OR cross-disciplinary:ti OR crossdisciplinary:ti OR multidisciplinary:ti OR multi-disciplinary:ti OR multi NEXT disciplinary:ti OR interdisciplinary:ti OR interdisciplinary*:ti OR inter-disciplinary:ti OR comprehensive NEXT healthcare:ti OR continuity NEXT of NEXT patient NEXT care:ti OR guided NEXT care:ti OR integrated NEXT care:ti OR managed NEXT care:ti OR care NEXT manage*:ti OR shared NEXT care:ti OR integrated NEXT healthcare:ti OR collaboratory:ti OR collaborative:ti OR transmural NEXT care:ti OR multiprofessional:ti OR multi NEXT professional:ti OR multi-professional:ti OR care NEXT team*:ab OR cross NEXT disciplinary:ab OR cross-disciplinary:ab OR crossdisciplinary:ab OR multidisciplinary:ab OR multi-disciplinary:ab OR multi NEXT disciplinary:ab OR interdisciplinary:ab OR interdisciplinary*:ab OR inter-disciplinary:ab OR comprehensive NEXT healthcare:ab OR continuity NEXT of NEXT patient NEXT care:ab OR guided NEXT care:ab OR integrated NEXT care:ab OR managed NEXT care:ab OR care NEXT manage*:ab OR shared NEXT care:ab OR integrated NEXT healthcare:ab OR collaboratory:ab OR collaborative:ab OR transmural NEXT care:ab OR multiprofessional:ab OR multi NEXT professional:ab OR multi-professional:ab | 22,350 |
| #12 | #6 OR #7 OR #8 OR #9 OR #10 OR #11 | 24,154 |
| Block 5: Combined search | | |
| #13 | #5 AND #12 | 822 |
