# Supplementary material for: The Effectiveness of Collaborative Care Interventions for the Management of Patients With Multimorbidity: Protocol for a Systematic Review, Meta-Analysis, and Meta-Regression Analysis
Source: JMIR Res Protoc. 2024 Aug 8;13:e58296. doi: 10.2196/58296 (PMC11342003; doi:10.2196/58296)
Supplement: Multimedia Appendix 2 [file resprot_v13i1e58296_app2.docx]

# Multimedia Appendix 2: Study selection process

**Identification of studies via other methods**

**Identification of studies via databases and registers**

Records identified from:

Reference searching (n=x)

Citation searching (n=x)

Records removed *before screening*:

Duplicate records removed (n=1475)

Records marked as ineligible by automation tools (n=x)

Records removed for other reasons (n=x)

Records identified from:

Databases:
The Cochrane Library (CENTRAL) (n=822)

PubMed (n=1840)

Embase (n=1771)

CINAHL (n=2297)

**Identification**

Records screened (n=x)

Records excluded

(n=x)

Reports not retrieved (n=x)

Reports sought for retrieval

(n=x)

Reports sought for retrieval

(n=x)

Reports not retrieved

(n=x)

**Screening**

Reports excluded:

Not collaborative care (n=x)

Wrong population (n=x)

No subgroup analysis (n=x)

Comparative is not usual care (n=x)

No relevant outcomes (n=x)

Wrong language (n=x)

Protocol (n=x)

Wrong study design (n=x)

Not full text (n=x)

Not peer reviewed (n=x)

Reports excluded:

Not collaborative care (n=x)

Wrong population (n=x)

No subgroup analysis (n=x)

Comparative is not usual care (n=x)

No relevant outcomes (n=x)

Wrong language (n=x)

Protocol (n=x)

Wrong study design (n=x)

Not full text (n=x)

Not peer reviewed (n=x)

Reports assessed for eligibility

(n=x)

Reports assessed for eligibility

(n=x)

Studies included in review

(n=x)

Reports of included studies

(n=x)

**Included**
